# Current perspectives of cancer-associated fibroblast in therapeutic resistance: potential mechanism and future strategy

**DOI:** 10.1007/s10565-019-09461-z

**Published:** 2019-01-24

**Authors:** Dhruba Kadel, Yu Zhang, Hao-Ran Sun, Yue Zhao, Qiong-Zhu Dong, Lun-xiu Qin

**Affiliations:** 1 Department of General Surgery, Huashan Hospital & Cancer Metastasis Institute, Fudan University, 12 Urumqi Road (M), Shanghai, 200040 China; 2 Cancer Metastasis institute, Fudan University, Shanghai, 200040 China; 3 Institute of Biomedical Sciences, Fudan University, 131 Dong An Road, Shanghai, 200032 China

**Keywords:** Cancer-associated fibroblast, Drug resistance

## Abstract

The goal of cancer eradication has been overshadowed despite the continuous improvement in research and generation of novel cancer therapeutic drugs. One of the undeniable existing problems is drug resistance due to which the paradigm of killing all cancer cells is ineffective. Tumor microenvironment plays a crucial role in inducing drug resistance besides cancer development and progression. Recently, many efforts have been devoted to understand the role of tumor microenvironment in cancer drug resistance as it provides the shelter, nutrition, and paracrine niche for cancer cells. Cancer-associated fibroblasts (CAFs), one major component of tumor microenvironment, reside in symbiotic relationship with cancer cells, supporting them to survive from cancer drugs. The present review summarizes the recent understandings in the role of CAFs in drug resistance in various tumors. Acknowledging the fact that drug resistance depends not only upon cancer cells but also upon the microenvironment niche could guide us to formulate novel cancer drugs and provide the optimal cancer treatment.

## Introduction

Various studies have already identified that the nature of tumor does depend not only upon the malignant cancerous cells themselves but also to their microenvironment components (Kalluri 2003). The constituents of tumor microenvironment provide the shelter as well as paracrine niche for cancer cells that fuel the neoplastic growth. It functions as safeguard to tumor cells either by providing the mechanical support or secreting various factors evading the therapeutic effect. The role of microenvironment in promoting tumor growth and metastasis has been studied to some extent in various cancers (Kalluri and Zeisberg 2006; Li et al. 2007; Tlsty and Coussens 2006) but its role in anti-cancer therapeutic resistance is still poorly understood (Shekhar et al. 2007; McMillin et al. 2010; Wang et al. 2009). Tumor microenvironment comprised of both pro-tumorigenic and anti-tumorigenic components such as stromal cells (normal fibroblasts, cancer-associated fibroblast (CAFs), immune inflammatory cells, endothelial cells, pericytes, bone marrow-derived cells, etc.), structural elements of extracellular matrix (ECM), and soluble factors (such as cytokines, growth factors) (Li et al. 2007; Tlsty and Coussens 2006; Quail and Joyce 2013). Recent researches have suggested that these elements interact with tumor cells as well as with each other forming a complex crosstalk network and create either tumor-prone or tumor-suppressive microenvironment, although the involved molecular mechanism is not well understood (Quail and Joyce 2013; Grivennikov et al. 2010; Palucka and Coussens 2016).

CAFs constitute major proportion of non-neoplastic stromal compartment in various human tumors. Various researches have suggested that they are capable of modulating tumor cells by forming the communication network with cancer cells or with other elements and hence susceptible to cancer drug resistance (Orimo and Weinberg 2006; Mueller and Fusenig 2004). So, focusing on both cancer cells and CAFs might provide some new hints for cancer treatment. Here we review the role of CAFs in cancer drug resistance, underlying molecular mechanisms as well as the approached strategies to overcome the potential resistance induced by CAFs.

### Origin and markers of CAFs

CAFs should be considered as the structural and functional alteration rather than cell type variation. Under the various intrinsic or extrinsic influential factors, structural and functional modifications on progenitor cells occur, which, to our current knowledge, are known as CAFs. The progenitor states are transformed to CAFs during the tumor progression, and some of the well-known progenitors are resident fibroblast and immune cells (Kojima et al. 2010; Erez et al. 2010), bone marrow-derived mesenchymal stem cells (Mishra et al. 2008; Quante et al. 2011; Spaeth et al. 2009; Jeon et al. 2008; Direkze et al. 2004), epithelial cells (Kalluri and Neilson 2003), endothelial cells (Zeisberg et al. 2007), hepatic stellate cells (Yin and Evason 2013), and pancreatic stellate cells (Jaster 2004). Cancerous cells attract bone marrow-derived MSCs to the tumor microenvironment and convert them into CAF-like myofibroblastic phenotype (Mishra et al. 2008; Bergfeld and Declerck 2010). These structurally altered CAFs, which formerly recognized as MSCs, then support tumor cell survival and angiogenesis, possess immunomodulatory function, and lead to drug resistance (Bergfeld and Declerck 2010). Moreover, the well-known resident fibroblasts of pancreas, pancreatic stellate cells, exhibit vitamin A containing lipid droplets in its quiescent state. Once communicated with tumor cells become activated and loose the vitamin A reserving potential, which then display contractile and secretory phenotype. The secretory function of these activated pancreatic stellate cells favors tumor survival (McCarroll et al. 2006).

CAFs are predominantly composed of activated fibroblast, but also with less amount of non-activated fibroblast (Shimoda et al. 2010; Hanahan and Coussens 2012). The activated fibroblast population in CAFs is identified by their expression of specific markers such as α-smooth muscle actin (α-SMA), vimentin, desmin, fibroblast activation protein (FAP) (Mueller and Fusenig 2004), fibroblast specific protein (FSP) (Strutz et al. 1995), platelet-derived growth factor receptor (PDGFR) (Pietras et al. 2003), secreted protein acidic and rich in cystein (SPARC), chondroitin sulfate proteoglycan (Sugimoto et al. 2006), prolyl-4 hydroxylase (Kojima et al. 2010), periostin (Malanchi et al. 2012), integrin alpha 11 (Zeltz and Gullberg 2016), and tenascin C (De Wever et al. 2004), where the expression of these markers varies from one cell to another, suggesting the existence of heterogenic population of CAFs. Among these markers, α-SMA showed large labeling pattern and has long been accepted as the most reliable marker for identifying activated fibroblast in CAFs (Sugimoto et al. 2006). On the other hand, progenitor states lack α-SMA expression and are transformed to α-SMA-positive CAFs via cancer cell-derived growth factors such as transforming growth factor-β (TGF-β) (Orimo et al. 2005), platelet-derived growth factor (PDGF) and fibroblast growth factor-2 (FGF-2) (Elenbaas and Weinberg 2001), Wnt7a (Avgustinova et al. 2016), sonic hedgehog (Shh) (Bailey et al. 2008), and exosomes (Paggetti et al. 2015; Webber et al. 2015). In contrast, there is evidence that leukemia inhibitory factor (LIF), a member of the interleukin-6 (IL-6) pro-inflammatory cytokine family, can transform progenitor state to pro-invasive fibroblast independently of α-SMA expression (Albrengues et al. 2014).

**Fig. 1:**
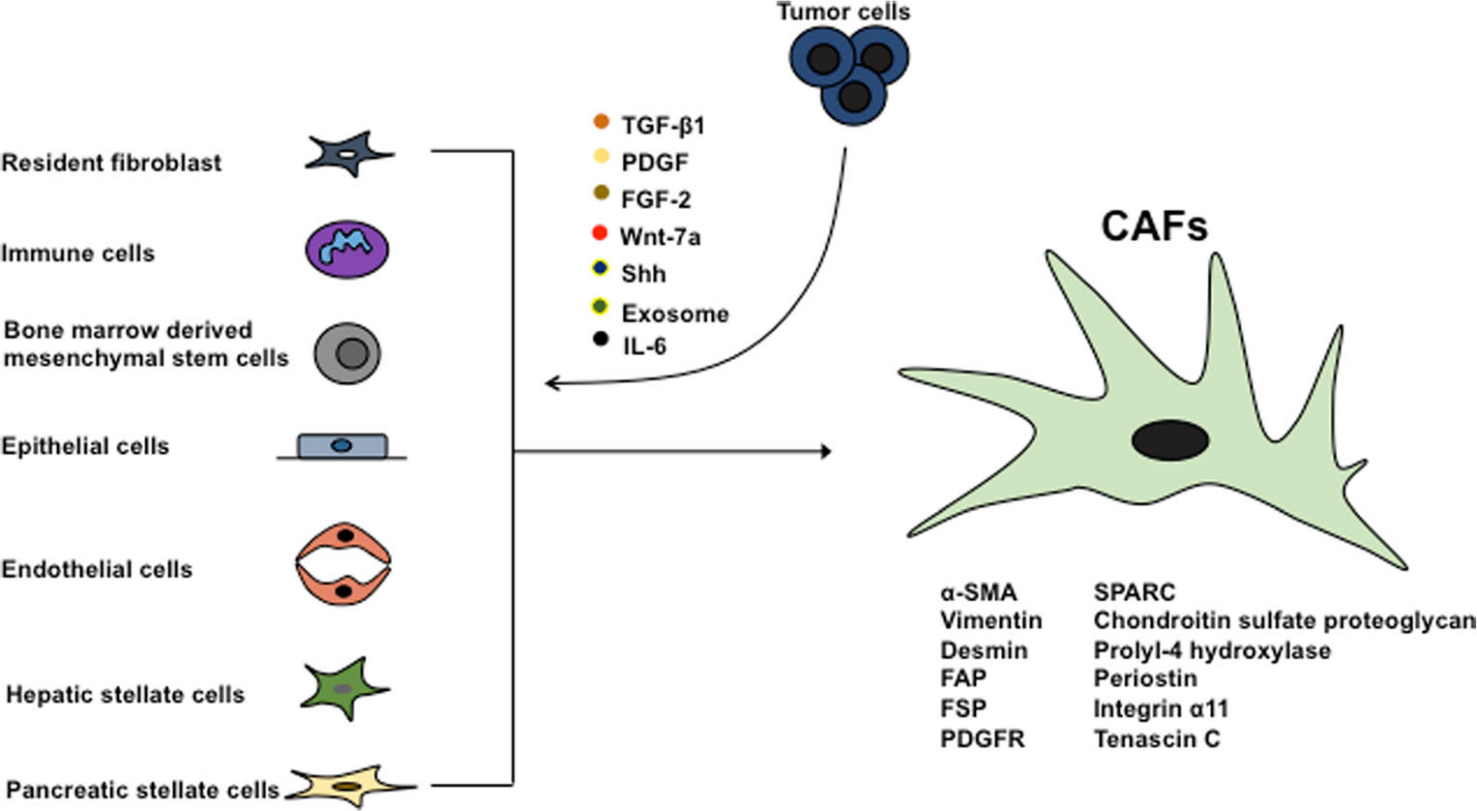
Heterogeneous origin of CAFs and its markers. Cancer-associated fibroblasts are originated from various sources, which are in quiescent state and convert into CAFs after communicating with malignant cells and express different markers differentiating from its progenitor state

Thus, the progenitor and quiescent precursor of CAFs contributes favorable tumor microenvironment to some extent for tumor survival when acquires phenotypic variation (Fig. 1). The tumor-promoting properties of CAFs such as proliferation, angiogenesis, invasion, and metastasis are regulated by various signaling pathways that include stromal cell-derived factor-1 (SDF-1)-[C-X-C] chemokine receptor-4 (CXCR4) and TGF-β-Smad2/3 (Kojima et al. 2010), JAK1/STAT3 (Albrengues et al. 2014; Sanz-Moreno et al. 2011; Albrengues et al. 2015), interleukin-1β (IL-1β)-nuclear factor kappa B (NF-κβ) (Erez et al. 2010), PDGF-PDGFR (Elenbaas and Weinberg 2001; Shao et al. 2000), Yes-associated protein/Tafazzin (YAP/TAZ) (Dupont et al. 2011; Calvo et al. 2013), Shh-Smoothened (Smo) (Bailey et al. 2008), P62-mTORC1 (Valencia et al. 2014), loss of Timp gene (Khokha et al. 2013), and global hypomethylation of genomic DNA (Hu et al. 2005; Jiang et al. 2008).

### CAFs in tumor suppression

Even though pro-tumorigenic properties of CAFs have been revealed by numerous studies, the inefficacy of stromal targeted therapies in several preclinical studies have raised the doubt in clinical application. A significant number of possible explanations have been put forwarded, and the interesting but insufficient evidences suggested the possibility of tumor-suppressive function of CAFs. The long-term administration of hedgehog (Hh) inhibitor in genetically engineered pancreatic ductal adenocarcinoma (PDAC) and chemically induced bladder cancer mice model showed reduction in stromal contents consequently promoting tumor growth and malignancy, which indicated stromal cells function as tumor suppression (Rhim et al. 2014; Shin et al. 2014). This finding was also supported by other study showing that the depletion of CAFs resulted in suppressed immune surveillance with increased CD4+FoxP3+ Tregs and led to invasive, poorly differentiated, and enhanced stemness of cancer cells experimented in PDAC mice model (Özdemir et al. 2014). Contradictory to many previous studies, a recent study verified that presence of higher number of FAP+ CAFs (> 100/high-power field) in PDAC stromal cells is associated with prolonged survival (Park et al. 2017). Similarly, the other study revealed the mechanism of CAFs in tumor suppression via Slit2-Robo1-mediated suppression of PI3K/AKT/β-catenin pathway in breast cancer cell lines (Chang et al. 2012). Another example of tumor-suppressive function of CAFs is that the deletion of nuclear factor kappa B kinase subunit β (IKKβ) in murine model of colitis-associated tumorigenesis resulted in neoangiogenesis and tumor progression showing the tumor-suppressive role of IKKβ (Pallangyo et al. 2015); however, opposite results were shown in the same model by other research group (Koliaraki et al. 2015). The contradictory results observed in two different studies with similar model could be explained as (1) IKKβ was deleted in type-I-collagen fibroblast by the first research group and in type-VI-collagen fibroblast by the latter one; (2) besides genetic background of the mice, timing of IKKβ deletion and (3) existence of heterogeneous fibroblast subpopulation in tumor stroma might play a role in different findings (Wagner 2016).

Inconsistent outcomes of CAFs in pro- and anti-tumorigenicity might be due to its wide sources as well as diversity in its secretory function and establishing different communication network with tumor cells. The origin of CAFs determining its nature could be illustrated as CD271+. Pancreatic stellate cells showed anti-tumorigenic properties (Fujiwara et al. 2012) while CD10+ pancreatic stellate cells were identified as pro-tumorigenic nature (Ikenaga et al. 2010). Whereas the secretory function characterizing CAFs was also shown by various studies. One study verified the dual function of periostin secreted by CAFs as its slight overexpression significantly reduced epithelial-mesenchymal transition (EMT), whereas higher overexpression enhanced EMT in human pancreatic cells (Kanno et al. 2008). Moreover, one of the major ECM factors secreted by CAFs is hyaluronan (HA), whose role in cancer has been studied well. Large number of clinical analysis have shown that tumor progression and poor outcome in various cancers is associated with higher accumulation of HA either in stroma or in tumor parenchyma (Wu et al. 2017; Turley et al. 2016; Sato et al. 2016; Chanmee et al. 2016; Bourguignon et al. 2017). Studies have shown that CAFs secreted HA played crucial role in migratory interaction of CAFs and tumor cells. So, the mitogenic properties of highly motile CAFs are based on HA concentration (Costea et al. 2013). Apart from promoting CAF motility, a major susceptible factor for cancer supporting role of HA might be its participation in EMT program. Studies have shown that HA accumulation either by HAS3 overexpression or by hypoxia inducible factor-1α (HIF-1α) leads to induction of EMT (Zhang et al. 2013; Misra et al. 2008; Kultti et al. 2014; Gao et al. 2005). However, some recent studies have recognized HA as tumor-suppressing stromal component (Bohaumilitzky et al. 2017; Fisher 2015; Triggs-Raine 2015; Tian et al. 2013). The cancer resistance properties of HA were first noted in naked rat mole, where authors observed that naked rat mole fibroblasts secreted extremely high molecular mass HA, cells possessed higher affinity to HA signaling, and HA-degrading enzyme showed less activities (Tian et al. 2013). The elaborative study pointed that high molecular HA has ability to hypersensitize cells to contact inhibition and stimulate p16 (INK4a locus) expression resulting in cell cycle arrest (Tian et al. 2015). Consistent to this, another study revealed that excess HA production was found to be associated with inhibition to G1-S transition in cell cycle rather than acting as tumorigenesis (Bharadwaj et al. 2011). Supporting to this, hyaluronidase showed tumor growth suppression and facilitated cancer drug treatment (Wong et al. 2017; Melanie and Simpson 2008). These observations can conclude that high-weight HA with less tendency for degradation could provide the cancer resistance properties.

**Fig. 2:**
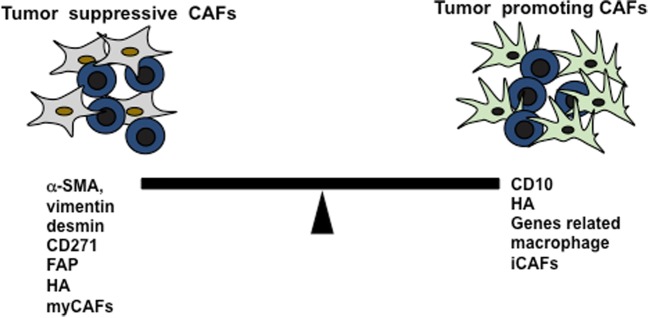
The dual nature of CAFs. Two distinct subpopulation of CAFs that are based on its secretory function and expression markers and play the subsequent role in tumor microenvironment

The dynamic nature of CAFs during the cancer progression focuses on further need for discussion to recognize it as friend or foe. In author’s view, categorizing the specific subpopulation of CAFs would more precisely characterize their role in cancer environment. A study identified two distinct subpopulations of CAFs as inflammatory fibroblast (iCAFs) and myofibroblast (myCAFs), where they observed that myCAFs lack tumor-promoting chemokines and cytokines (Öhlund et al. 2017). Furthermore, another study also identified the two distinct subpopulation of stromal types based on gene expression as normal (expressing high CAF markers such as α-SMA, vimentin, and desmin) and activated (expressing more gene-related macrophage) stroma (Moffitt et al. 2015), as shown in Fig. 2. This can hypothesize that normal stromal cell can be considered as good stroma and may show tumor-suppressive function. Combining the aforementioned studies, a detail study can be carried out to identify if good stroma only secretes high molecular mass HA. Thus, it alarms the preservation of tumor-suppressing stromal cells when generating stromal-targeted therapies for cancer treatment.

## Roles of CAFs in drug resistance

### Drug resistance via revascularization and reactivating MAPK and Akt

One of the problems in eradication of cancer is the drug resistance. Tumor cells follow different paths to become resistance to cancer treatment depending upon the intrinsic properties or external stimuli from microenvironment (Hata et al. 2016). Many researchers are focusing on various external stimuli from tumor microenvironment inducing drug resistance. Numerous studies have tried to explore the role of stromal cells, especially fibroblasts and CAFs in drug resistance in different tumors (Straussman et al. 2012; Mao et al. 2013; Paraiso and Smalley 2013). Majority of studies have proven that the secretory function of CAFs, which establishes the crosstalk with tumor cells, is responsible for drug resistance. One study revealed that CAFs extracted from anti-VEGF resistance murine lymphoma cell line (EL4) could effectively resist the anti-VEGF therapy in otherwise sensitive murine myeloma cell line (TIB6) via platelet-derived growth factor-C (PDGF-C) mediation (Crawford et al. 2009). Authors observed the upregulation of PDGF-C in CAFs and hence inducing the revascularization, which ultimately led to anti-angiogenic therapy resistance. Most of the patients treated with anti-angiogenesis such as sorafenib, sunitinib, and bevacizumab appeared to be low/no response after certain period of treatment with only modest benefit in clinical outcomes (Jayson et al. 2016). This could suggest that there might be morphologic and functional changes of CAFs after exposure to anti-angiogenic treatment recreating the angiogenic environment and becoming refractory to drug therapy. Another experiment showed that stromal fibroblast could induce resistance to epidermal growth factor receptor (EGFR) tyrosine kinase inhibitors via hepatocyte growth factor (HGF) mediated crosstalk between tumor cells and stromal cells in lung cancer (Wang et al. 2009). Furthermore, the study carried out in tumor microenvironment induced drug resistance showed that various BRAF mutated melanoma and ERBB2+ breast cancer cell lines when co-cultured with different fibroblasts affected the sensitivity to vemurafenib and lapatinib, respectively, via HGF/c-MET pathway (Straussman et al. 2012). This result was also supported by another study revealing that most cancer cells could be rescued from drug by simply exposing to one or more receptor tyrosine kinase (RTK) ligands (Wilson et al. 2012). It is well known that HGF/c-MET signaling activates MAPK cascades enhancing cancer cell proliferation and Akt cascades increasing the anti-apoptotic effects, which could eventually increase the drug tolerance capacity in various cancers (Xiao et al. 2001).

### Drug resistance in hypoxic environment

Many studies have pointed out the ability of CAFs to modulate drug sensitivity in hypoxic environment. CAFs secrete different angiogenic factors and prominent one being the VEGF, whose production is even increased in hypoxic state (Beckermann et al. 2008; Masamune et al. 2008). However, a recently published study verified that endothelial cell sprouting was observed more with hypoxic CAFs even upon blocking VEGFA, which indicated presence of other angiogenic agent (Kugeratski et al. 2016). Further study on proteomic analysis of hypoxic CAFs revealed the upregulation of proteins related to glycolysis and downregulation of proteins related to mitochondrial metabolism. A detail study is needed to unveil the other associated factors responsible for angiogenesis. Furthermore, hypoxia stimulated pancreatic fibroblast and promoted angiogenesis via expression of VEGF receptors, angiopoetin-1 and Tie-2, and these pancreatic fibroblasts induced pancreatic cancer cell motility via IGF1/IGF1R signaling (Masamune et al. 2008; Hirakawa et al. 2016).

Acidic environment created by hypoxia produces the lactic acid within ECM without changing the intracellular PH that blocks the accumulation of drugs within the cancer cells (Harrison and Blackwell 2004; Vukovic and Tannock 1997). In some drugs such as doxorubicin, which is oxygen-dependent, effect is obviously reduced in hypoxic environment (Harrison and Blackwell 2004). Hypoxic environment drives the hypoxia-inducible factor 1 (HIF-1) to upregulate the drug resistance genes encoded ATP-binding cassette transporters (ABC) (Wartenberg et al. 2003). Proteins of ABC family have been well studied and their functions are well known, which play a major role in drug resistance through multiple functions including efflux of drug from cancer cells (Fletcher et al. 2010). Moreover, as mentioned previously, HIF-1α signaling is associated with HA accumulation promoting EMT and ultimately to drug resistance (Zhang et al. 2013; Gao et al. 2005). A study pointed out the possible way to overcome this by depleting HA accumulation, which inhibits HIF-1α-snail signaling and suppresses EMT (Kultti et al. 2014). The role of hypoxia in generating the tumor drug resistance has been well reviewed and suggested to target hypoxia in treating cancer to get the better outcome (Wilson and Hay 2011). These studies provided the sufficient proofs that the regulation of drug sensitivity of cancer cells by CAFs, especially in hypoxic environment, is also inevitable. However, hypoxic CAFs secreted key player to induce the resistance may not have been characterized yet and continuous effort is needed to unravel it.

Contradictory to this, a study reported that the prolonged hypoxia can cause the deactivation of CAFs and diminishes the role of CAFs (Madsen et al. 2015). A detail study is needed to clarify it more.

### Drug resistance by secreting soluble factors

As mentioned earlier, tumor microenvironment, notably fibroblasts and CAFs, elicits drug resistance via secretion of various soluble factors. Among these proteins, Wnt family member wingless-type MMTV integration site family member 16B (WNT16B), which is regulated by NF-κβ after DNA damage, activates the canonical Wnt program in tumor cells and induces cytotoxic chemotherapy resistance in prostate cancer (Sun et al. 2012). They showed that WNT16B was upregulated in chemotherapy administered prostate, breast, and ovarian cancers and verified both in vitro and in vivo that WNT16B secreted by fibroblast was responsible for inhibiting the chemotherapy-induced apoptosis. Another study revealed that fibroblasts secreted frizzled-related protein 2 (SERP2) after genotoxic treatment could augment β-catenin activities initiated by WNT16B and enhanced chemotherapy resistance in prostate cancer (Sun et al. 2016). Moreover, other proteins, cytokines and chemokines, secreted by CAFs can switch the tumor cells genotypically and phenotypically exhibiting the drug resistance properties. Interleukin-17A (IL-17A) secreted by chemotherapy-treated human CAFs promoted the colorectal cancer initiating cell (CIC) self-renewal and tumor growth displaying the conventional chemotherapy resistance features (Lotti et al. 2013). Authors noted the significant increase of CAFs after the chemotherapy, which further suggested the possibilities of alteration of CAF features induced by various drugs. This speculation was supported by another study showing that initially well responded BRAF-mutant melanoma cells to PLX4720 became tolerant after certain period of treatment by reactivating ERK/MAPK in the areas of high stromal density (Hirata et al. 2015). This is due to activation of stromal fibroblast, elevation of matrix production, and remodeling leading to elevated integrin β1/FAK/Src signaling in melanoma cells associated with BRAF inhibitor, PLX4720. These findings suggested that chemotherapy or radiotherapy could enhance the secretions of stromal derived factors besides killing cancer cells, and provide the survival benefit to cancer cells and resulting in drug resistance. This concept was also supported by other two independent studies (Acharyya et al. 2012; Nakasone et al. 2012). Therefore, approaches to curative treatment to cancer became likely insufficient due to avoiding the fact of therapy-derived functional switch of stromal cells.

### Drug resistance via stromal modification

Desmoplastic stroma also could sufficiently hinder the drug delivery by inducing vascular collapse. A study in PDAC mouse model refractory to gemcitabine was observed poor vascularization with poor perfusion, but when co-administered with IPI-926, hedgehog cellular signaling inhibitor, significantly increased both vascularization and perfusion by reducing tumor-associated stromal tissue (Olive et al. 2010). Moreover, HA was also found to be responsible for generating high interstitial fluid pressure (IFP) inducing vascular collapse and acted as barrier for drug perfusion and diffusion in PDAC mice model (Provenzano et al. 2012). Researchers found that enzymatic breakdown of stromal hyaluronic acid before administration of cancer drug therapy resulted in remodeling of microenvironment, normalized IFP, and reestablished the microvascular structure, which ultimately enhanced the therapeutic effect. This study was further supported by the research published in following year, observed that stromal hyaluronic acid depletion by PEGylated recombinant PH20 hyaluronidase (PEGPH20) tested in PDAC mice model could successively increase the intratumoral delivery of chemotherapeutic drugs, doxorubicin and gemcitabine (Jacobetz et al. 2013). Apart from HA, CAFs also modulated the drug delivery in cancer cells via mechanism of PDGFR involved increased IFP (Heldin et al. 2004). The role of PDGFR in drug resistance by increasing IFP have been supported by other independently published data showing that PDGFR inhibitor could reduce the vascular intercellular pressure and facilitate the drug transport into the cancer cells (Pietras et al. 2003; Östman and Heldin 2007). These findings suggested that desmoplastic stroma and hypoperfused tumor generated by CAFs could hinder the effective drug perfusion leading to drug resistance. Moreover, matrix remodeling by CAFs can form the chemoprotective niche and hence blocks the effective delivery of chemotherapeutic drugs to the cancer cells via cell adhesion-mediated drug resistance (CAM-DR), contributing its role in evading cancer cells to treatment (Meads et al. 2009). Researchers have described the presence of number of molecules on the cell membrane of CAFs such as cell surface proteoglycans, integrin, and non-integrin collagen receptors, which participate in cell adhesion mechanism that protect cancer cells from drug-induced apoptosis (Zeltz and Gullberg 2016; Multhaupt et al. 2016). One study explained the role of CAM-DR on modulation of cancer cell adhesion on ECM in multiple myeloma showing that the adhesion resulted in increased p27kip1 levels that associated with cell cycle arrest and resistance to melphalan (Hazlehurst and Dalton 2001). The role of focal adhesion molecule including integrins, integrin-associated proteins, and growth factor receptors have been highlighted in recently reviewed article (Eke and Cordes 2015). These findings suggested that cell-matrix interaction caused reorganization of cytoskeleton and induced the multiple signaling pathways, which was sufficient to cause drug resistance. However, the experiment on mouse model of pancreatic cancer showed that desmoplastic response and fibrosis could be restored by the inhibition of Hh-Smo pathway and permitted the well distribution of drugs in the cancer cells (Olive et al. 2009). So, there is still dim hope to overcome the cancer drug resistance due to stromal modification, and continue study to understand the mechanism in detail is needed.

### Role in endocrine treatment resistance

The endocrine treatment resistance is widely observed even in estrogen receptor-positive (ER+) breast cancers. A research performed in ER+ MCF-7 cells showed that CAF was the source of tamoxifen and fulvestrant resistance and also protected cancer cells from doxorubicin and PARP inhibitor (Martinez-outschoorn et al. 2011). They found that tamoxifen promoted the upregulation of TP53-induced glycolysis and apoptosis regulator (TIGAR), P53 regulated gene that can inhibit glycolysis, autophagy, and apoptosis and reduces ROS generation, in CAF co-cultured MCF-7 cells enhancing the oxidative mitochondrial metabolism, which provided the survival benefit to cancer cells. The inhibition of mitochondrial activities by metformin or arsenic trioxide led to increase in glucose uptake by mitochondria resulting in metabolic imbalance between cancer cells and CAFs that resensitized tamoxifen treatment. Another independent research on MCF-7 cells showed that soluble factors secreted by fibroblast rescued the tumor cells from tamoxifen by the mechanisms that involved EGFR and matrix metalloproteinases (Pontiggia et al. 2012)]. They verified that stromal factors as the modulators of ER activity showing that fibroblasts were able to phosphorylate ER at serine-118. In contrast to this, a research performed by Shekhar et al. showed that sensitive premalignant EIII8 and tumorigenic MCF-7 cells when co-cultured with the fibroblast derived from ER−/progesterone receptor (PR)− human breast tumors conferred the tamoxifen resistance independently of EGFR (Shekhar et al. 2007). They observed the non-correlation between tamoxifen resistance and level of EGFR or phospho-EGFR and endocrine sensitivity. However, some studies have concluded that the in vitro EGFR sensitivity in neck squamous cell carcinoma and lung cancer is modulated by co-cultured fibroblast, indicating that the role of fibroblast in cancer drug resistance via EGFR mechanism should not be neglected (Wang et al. 2009; Johansson et al. 2012).

### Role in immunotherapy resistance

The hope to treat cancer patients is becoming dim with the increased report of immunotherapy resistance. Immune system is complex to understand but various researches have tried to explore the role of immune system in cancer development and put forward the idea of dual function of immune cells, pro- and anti-tumor progression (DeNardo et al. 2010). In fact, the role of microenvironment components in tumor promotion or suppression somehow depends on the nature of infiltrated immune cells in the microenvironment (Ostrand-Rosenberg 2008). Tumor microenvironment is infiltrated by various immune cells in response to inflammation and secret growth factors such as TGF, FGF, VEGF, and some members of the interleukin family, which can act as driving force for fibroblast activation (De Visser et al. 2006; Calvo and Sahai 2011). In the other hand, fibroblasts are also able to modulate the tumor immune system. The role of fibroblast in altering the immune-controlled tumor growth was described in transgenic Lewis lung carcinoma and subcutaneous PDAC mouse model, where it was observed that depletion of FAP-expressing cells enhanced the hypoxic necrosis of both tumor and stromal cells by a process involving interferon-γ (IFN-γ) and tumor necrosis factor-α (TNF-α) (Kraman et al. 2010). This result was further supported by another research showing FAP-expressing CAF as the main source for the resistance of two immunological checkpoint antagonists: anti-cytotoxic T lymphocyte-associated protein-4 (α-CTLA4) and α-programmed cell death ligand 1 (α-PD-L1), in PDAC mice model (Feig et al. 2013). The failure of these checkpoint inhibitors was driven by CAF-secreted chemokine [C-X-C motif] ligand 12 (CXCL12). Administration of CXCL12 receptor inhibitor enhanced the T cell accumulation at cancer site and acted synergistically to those immunological checkpoint antagonists. These research findings alarmed that CAFs are consistently supporting cancer cells to evade a potential curative therapy of cancer, immunotherapy, requiring to further explore the function of CAFs.

#### Drug resistance via epigenetic modification

Various researches have pointed that the epigenetic modification of cancer cells induced by CAFs is responsible for drug resistance. Some studies have reported that DNA methylation status of stroma associated with the methylation profile of adjacent malignant cells (Hu et al. 2005; Hanson et al. 2006). Consistent to this, soluble factors secreted by CAFs upregulated wide patterns of genes (372 genes) in breast cancer cells that are epigenetically modified by DNA hypermethylation at transcription start site and shore regions. This effect was silenced on inhibition of DNA methylation (Mathot et al. 2017). This was further supported by another research showing that CAF secreted factors were responsible for combinatorial DNA hyper/hypomethylation, which induced EMT and stemness phenotype in prostate cancer cell (Pistore et al. 2017). They observed that methylation was DNM3TA dependent and its knockdown prevented EMT program. Many researches have already verified that EMT and stemness were also one major contributory factor for cancer drug resistance. Furthermore, a study revealed that EMT and metastasis were promoted by TGF-β secreted by CAFs, which catalyzed the global DNA hypermethylation changes in epithelial ovarian cancer cell (Cardenas et al. 2014). Authors further observed that DNMT inhibitor knock down the hypermethylation and EMT effect.

Similarly, few studies are available showing that posttranscriptional histone modification of cancer-associated CAFs caused cancer-promoting behavior. A study showed that expression of both the histone mark H3K27me3 and enhancer of zeste homolog 2 (EZH2) was decreased in breast CAFs and promoted cancer invasion by overexpression of thrombospondin type 1 (Bracken et al. 2006; Tyan et al. 2012). Epigenetic modification on cancer cells induced by CAFs causing drug resistance is outgrowing topic, and a detailed study is needed to address the drug resistance issues. In our knowledge, a better result could be expected by combination of demethylating agent and targeting agent to CAF secreted factor responsible for epigenetic modification.

#### Anti-fibroblastic therapies

Many studies are available examining the anti-fibroblastic therapies in cancer treatment. Analyzing the various related published research work, the current focus being either to deplete the stroma or inactivate CAFs. The most potential one is targeting to HA, stromal component secreted by CAFs. A phase Ib clinical study showed that doxorubicin increased median progression-free survival and overall survival when applied in combination with PEGPH20 (7.2 and 13 months in high HA and 3.5 and 5.7 months in low HA tissue level, respectively) (Hingorani et al. 2016). This was in consistent with another phase II study, which showed that nab-paclitaxel/gemcitabin in combination with PEGPH20 obviously increased progression-free survival in patients with metastatic PDAC as compared to nab-paclitaxel/gemcitabine [hazard ratio (HR) 0.73, *P* = 0.049 and in patient with high HA tumors HR 0.51, *P* = 0.048] (Hingorani et al. 2018)]. As mentioned previously, PEGPH20 degraded HA and nab-paclitaxel also believed to be depleting agent of stromal component (Jacobetz et al. 2013; Von Hoff et al. 2013). Similarly, a recently published article showed the increased concentration of carboxymethyl cellulose docetaxel nanoparticle (CellaxTM-DTX) at SMA CAFs degrading the stromal component. Whereas, the latest studies have also verified the possibility of reverting activated CAFs. A study showed that the anti-cancer compound minnelide, water-soluble pro-drug of triptolide extracted from Chinese plant *Tripterygium wilfordii*, disrupted TGF-β signaling and hence reverted activated CAFs to quiescent form by decreasing α-SMA expression, reducing ECM secretion, and increasing vitamin A-containing lipid droplets (Dauer et al. 2018)]. Moreover, another study also supported the strategy to revert activated CAFs showing that application of all-transretinoic acid (ATRA) promoted the quiescence form of pancreatic stellate cells resulting in Wnt-beta-catenin signaling and reduced tumor progression (Froeling et al. 2011).

Thus, newly recognized CAFs targeted molecules like PEGPH20, nab-paclitaxel, CellaxTM-DTX, minnelide, and ATRA could be regarded as the novel cancer therapeutic agent in the modern era. However, it should also be noted that the various researches have pointed that simple depletion of stroma could promote spreading of cancer cells rather than only facilitating drug delivery. A study reported that simple blocking of serum amyloid A1 (SAA1), poor prognostic marker in pancreatic cancer cells, did not inhibit tumor growth and they verified two subtypes: saa3-competent and saa3-null CAFs, which showed pro-tumorigenic and anti-tumorigenic effect, respectively (Djurec et al. 2018). So, in author’s view, selective depletion of stroma would provide the better result in cancer treatment.

As mentioned previously, one reason behind the resistance of a new hope of cancer treatment, an immunotherapy, is induced by CAFs. But, dim hope still remains with continuous exploration and understanding its mechanism. CAF-induced resistance of two checkpoint inhibitors (α-CTLA4 and α-PD-L1) was blocked by CXCL12 receptor inhibitor (Feig et al. 2013)]. Similarly, Janus Kinase 2 (JAK2) inhibitor, which can modify the stroma, also decreased PD-L1 expression and enhanced the anti-cancer treatment as demonstrated in in vitro cancer cells (Wörmann et al. 2016; Doi et al. 2017)]. Consistent to this, another research showed possible enhancement of anti-PD-L1 treatment via inhibiting the IL-6, a JAK activator (MacE et al. 2018). Hence, all these evidences verify that targeting stroma can enhance the immunotherapy.

## Discussion

**Fig. 3:**
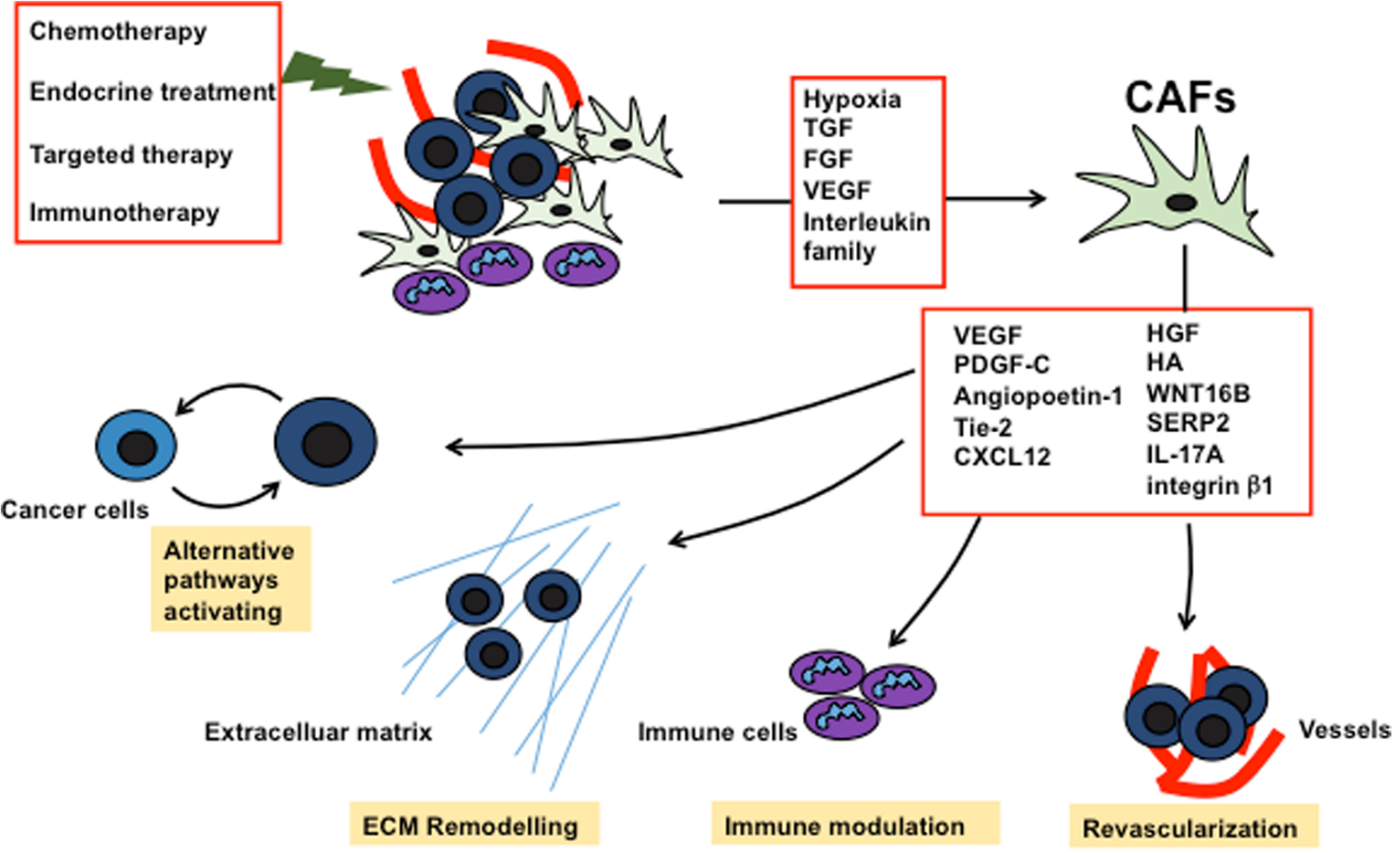
Roles of CAFs in drug resistance. CAFs play important role in cancer drug resistance via secretion of different factors and modulation of tumor microenvironment resulting reduction of drug efficacy or activation of cancer cells by alternating pathway

Despite the continuous progress in current cancer research and development of novel drugs, the problems against cancer drug resistance were not vanquished. Earlier, great efforts were given to resolve the cancer drug resistance focusing on the properties of cancer cells themselves, but recently, the role of microenvironment in drug resistance becomes the emerging topic for many researchers. Among the microenvironment components, CAFs have been long studied in exploring the mechanism of initiation of cancer, cancer progression, and metastasis (Kalluri and Zeisberg 2006). As discussed previously, CAFs should be regarded as the transitioned state rather than distinct cell type, but instead of knowing progenitors of CAFs, we are still unable to find the distinctive markers that can differentiate between different states. CAFs reside in the microenvironment as symbiotic relationship with cancer cells and provide the shelter, nutrition, and paracrine niche for tumor growth and function as backbone of microenvironment. Breakdown of this correlation is warranted to defeat cancer, and this can be achieved by understanding the structure and function of each of the constituents of microenvironment. Here we have reviewed the role of CAFs, pillar of tumor microenvironment, in drug resistance, and its underlying mechanism and highlighted the feasible way of reversal. As shown in Fig. 3, we have tried to summarize the CAF’s contribution in drug resistance by different aspects such as participating in revascularization, immune modulation, ECM remodeling, and providing the alternative pathway for cancer cells to induce drug resistance. Knowing these facts would help in determining the better strategy for the treatment of cancer patients and provides certain guidelines in the generation of next cancer drugs avoiding potential risk of therapy resistance.

Some of the researches have also pointed out the tumor-suppressive function of CAFs, which has been briefed above in this review, and suggested no need to pursue them while treating cancer to get the better outcome. The controversial study published regarding the tumor-suppressive role of CAFs has been discussed in detail in respective segment. But to our knowledge, failure to recognize CAFs as friend or foe is due to (1) unable to identify its specific origin (for, e.g., differences in CD271+ and CD10+ pancreatic stellate cells originated CAFs) (Fujiwara et al. 2012; Ikenaga et al. 2010), (2) inadequate knowledge to characterize its secreted factors (for, e.g., different function of CAFs secreted high- and low-weight HA), and (3) improper characterization of CAFs (for, e.g., existence of different subpopulation of CAFs as iCAFs and myCAFs). Moreover, the existence of heterogenic properties of CAFs also keeps us in dilemma in its appropriate characterization, which can be further elaborated as, as described above, CAFs are able to express various markers, and none of the markers are completely overlapped; CAFs’ contents are relatively higher in highly dense breast, pancreatic, and prostate cancer while lower contents are detected in brain and renal tumors (Prakash 2016). CAFs are the phenotypic and functional switch from normal stromal cells, also termed as progenitors of CAFs within this review, and study reported that CAFs are much more competent in supporting tumor growth and metastasis than normal stromal fibroblasts (Orimo et al. 2005). One research has pointed out the importance of functional state of stromal cells, where they showed that the stromal signatures were more reliable than whole tissue signatures for predicting the clinical outcome in breast cancer patients (Finak et al. 2008). Drug sensitivity of cancer cells in the presence of CAFs also depends on the cancer types and microenvironment (Sonnenberg et al. 2008). Further study regarding identification and classification of CAFs is needed to better explain these existing problems.

One previous study showed that despite the heterogeneity of CAFs, they are genetically more stable than cancer cells. Therefore, targeting CAFs in cancer treatment would have smaller possibilities to develop drug resistance (Kerbel 1997)]. Recently published data have suggested the strategies to modulate CAFs either by inhibiting the activation pathway of CAFs or by breaking the drug delivery barrier created by CAFs. Preclinical studies showed that inhibiting TGF-β, angiotensin receptor, and Hedgehog signaling could reduce CAFs and ECM contents and improve the drug delivery (Olive et al. 2009; Liu et al. 2012). Similarly, suppression of CAF mediated secretion of IL-6 by inhibiting mTOR pathway via somatostatin analogue reversed the chemoresistance in pancreatic tumor (Duluc et al. 2015). Moreover, one recently published article showed that application of micro-RNA (miRNA) was possible in reverting the CAF phenotype to non-CAF phenotype (Kuninty et al. 2016). Detail discussion of targeted stromal therapy has been discussed in this review. To our knowledge, reversing the phenotype of CAFs rather than depleting may provide better solution for drug resistance.

In conclusions, CAFs, the building block of microenvironment, have a crucial role in modulating cancer drug sensitivity and understanding in detail could minimize the existing challenge of drug resistance and direct the future innovation of cancer drug by avoiding or overcoming the drug resistance.
